# The Use of Methotrexate and Mifepristone for Treatment of Interstitial Pregnancies: An Overview of Effectiveness and Complications

**DOI:** 10.3390/jcm12237396

**Published:** 2023-11-29

**Authors:** Davide Dealberti, Simona Franzò, David Bosoni, Carla Pisani, Victor Morales, Ivan Gallesio, Matteo Bruno, Giuseppe Ricci, Stefania Carlucci, Guglielmo Stabile

**Affiliations:** 1 Department of Obstetrics and Gynecology, “SS Antonio e Biagio e C. Arrigo Hospital”, 15121 Alessandria, Italy; ddealberti@ospedale.al.it (D.D.); dd.bosoni@gmail.com (D.B.); cpisani@ospedale.al.it (C.P.); victor.morales@ospedale.al.it (V.M.); 2 Department of Medicine, Surgery and Health Sciences, University of Trieste, 34100 Trieste, Italy; simona.franzo@burlo.trieste.it (S.F.); giuseppe.ricci@burlo.trieste.it (G.R.); 3 Research Center for Reproductive Medicine, Gynecological Endocrinology and Menopause, IRCCS S. Matteo Foundation, Department of Clinical, Surgical, Diagnostic and Pediatric Sciences, University of Pavia, 27100 Pavia, Italy; 4 Department of Radiology, Azienda Ospedaliera, “SS Antonio e Biagio e C. Arrigo Hospital”, 15121 Alessandria, Italy; igallesio@ospedale.al.it; 5 Department of Woman, Child, and Public Health, Fondazione Policlinico Universitario A. Gemelli IRCCS, 00168 Rome, Italy; brunomatteo2@gmail.com; 6 Institute for Maternal and Child Health IRCCS “Burlo Garofolo”, 34100 Trieste, Italy; s.carlucci86@gmail.com

**Keywords:** interstitial pregnancy, ectopic pregnancy, medical treatment, mifepristone, methotrexate, complications

## Abstract

Interstitial pregnancy is an unusual and potentially life-threatening form of ectopic pregnancy, accounting for approximately 1–6% of all ectopic pregnancies, with a maternal mortality rate of 2–2.5%. Implantation happens in the proximal portion of the fallopian tube as it passes through the myometrium. The resolution of interstitial pregnancy after medical treatment should be assessed by a decline in serum β-hCG, which occurs in about 85–90% of cases. Nonetheless, its effectiveness and consequences have been presented through case reports and case series. However, few cases of interstitial pregnancies treated totally medically with the use of methotrexate and mifepristone have been presented in the literature. Complications of this medical treatments have also never been reviewed before. In the present manuscript, we present a case of interstitial pregnancy treated with methotrexate and mifepristone. The patient after treatment developed a uterine arteriovenous malformation, treated with uterine artery embolization. Furthermore, we performed a systematic review of the literature using Scopus, PubMed and Google Scholar. A total of 186 papers were found, and 7 papers which included 10 cases were assessed for eligibility. The systemic medical treatment with the use of methotrexate and mifepristone was effective in 7 of the 10 cases. Two cases of hemoperitoneum following combined methotrexate and mifepristone treatment were reported. The applicability of this medical conservative treatment should be tailored to the patient, taking into account their obstetric history, gestational age at diagnosis and desire for future pregnancies. Complete resolution after this treatment was achieved in most of the cases reported without major complications. The appearance of uterine arteriovenous malformation can be managed conservatively, and we propose uterine artery embolization as an effective treatment of this rare complication.

## 1. Introduction

Interstitial pregnancy (IP) is an unusual and potentially life-threatening form of ectopic pregnancy (EP), accounting for approximately 1–6% of all EPs, with a maternal mortality rate of 2–2.5% [1]. Implantation happens in the proximal portion of the fallopian tube as it passes through the myometrium [2]. The use of assisted reproduction techniques, previous tubal surgery or previous ectopic pregnancies are considered predisposing factors to an interstitial pregnancy [3].

Historically, radical surgical treatment by laparoscopy or laparotomy was considered the standard procedure for interstitial pregnancies, ranging from an exploratory laparotomy with cornual wedge resection to a total abdominal hysterectomy [4,5]. Conservative surgical management includes laparoscopic resection of the pregnancy and involved tube with preservation of the uterine architecture [6]. Uterine artery embolization could be a therapeutic tool but only if associated with a conservative surgical approach (to reduce the risk of bleeding during the surgical procedure) or with a medical treatment using methotrexate [7]. A hysteroscopic laparoscopy-guided intervention, with or without embolization, has been proposed [8]. Close observation can also be proposed in extremely select cases when a patient is asymptomatic and has a spontaneously declining serum β-hCG. Conservative medical management has been proposed and used in the last years [9] and is now considered a promising alternative to surgery, especially in cases where fertility preservation is a priority, such as in young women who have not yet completed their family plan. Early diagnosis allows for the avoidance of surgery, which can result in hysterectomy and is burdened by the risk of complications. Patient selection for medical conservative treatment should include hemodynamical stability, early gestational age at diagnosis, low β-hCG serum levels, absence of contraindications to medical therapy, and patient preference and adherence to follow up [10]. Different drugs and methods of administration have been proposed over time. The administration of intramuscular methotrexate (MTX) (50 mg/m^2^) administered locally or systemically, in a single- or multidose regimen, has been deemed the most effective and tolerable option. The combination of MTX with a single dose of oral mifepristone (600 mcg) was shown to be successful [11,12,13]. Resolution of an interstitial pregnancy after medical treatment should be assessed by a decline in serum β-hCG, which occurs in about 85–90% of cases [14].

Nonetheless, its effectiveness and consequences have been presented through case reports and case series. However, few cases of interstitial pregnancies treated totally medically with the use of MTX and mifepristone have been presented in the literature. Complications of this medical treatment have also never been reviewed before. Evidence based on randomized studies is lacking due to the rarity of the condition.

In the present manuscript, we present a case of interstitial pregnancy treated with MTX and mifepristone. This is the case of interstitial pregnancy with the third-highest β-hCG treated with this approach. After considering all treatment options, including both conservative and surgical treatments, we chose this management, considering the experiences in the literature, the gestational age at diagnosis, the patient’s stable hemodynamic state, the size of the mass, the absence of abdominal free fluid, the absence of hepatic, renal and hematological impairment, and the patient’s desire to preserve their fertility. The patient after treatment developed a uterine arteriovenous malformation (AVM), subsequently treated with uterine artery embolization. This is the first time this kind of complication has been described. Furthermore, we performed a systematic review of the literature using Scopus, PubMed and Google Scholar to deepen our research and better understand what the results of medical treatment with MTX and mifepristone are. We would like to contribute to the existing literature with our experience, also presenting the management of any complications deriving from this type of medical therapy.

## 2. Case Report

**Figure 1:**
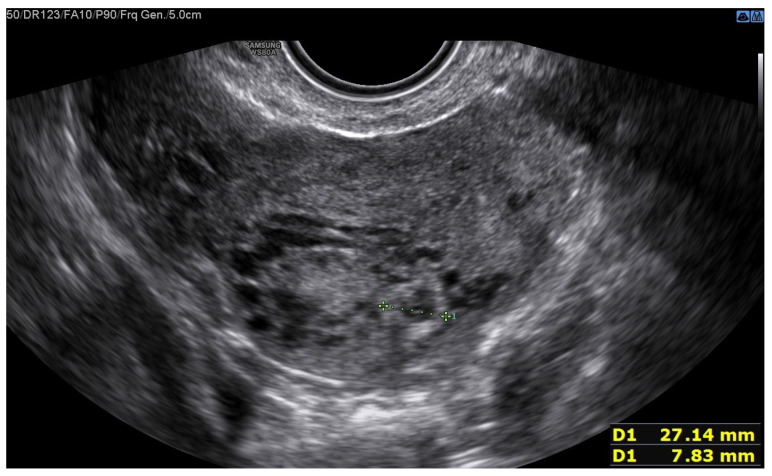
The green line identifies the gestational sac and a 2 mm embryo without a heartbeat.

**Figure 2:**
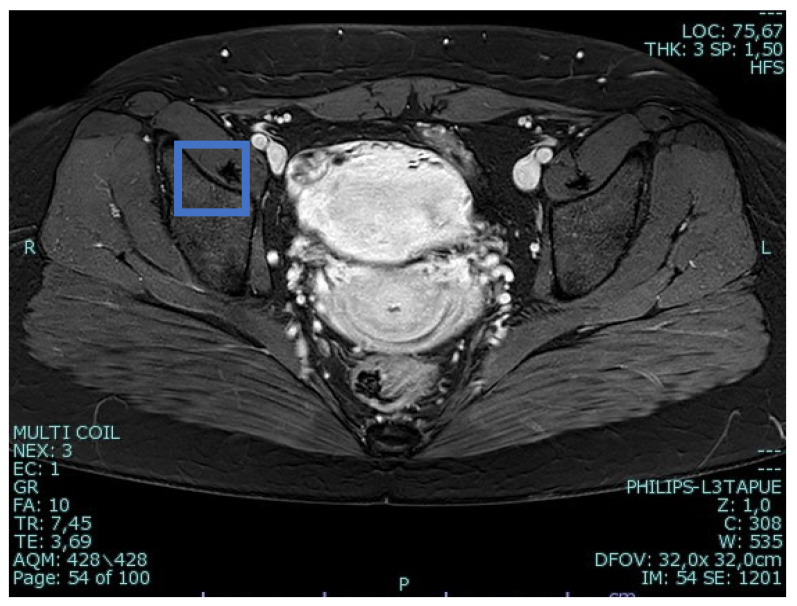
Pelvic nuclear magnetic resonance confirming the presence of an intramyometrial T2-hypointense signal alteration of 20 mm in the right cornual area.

**Figure 3:**
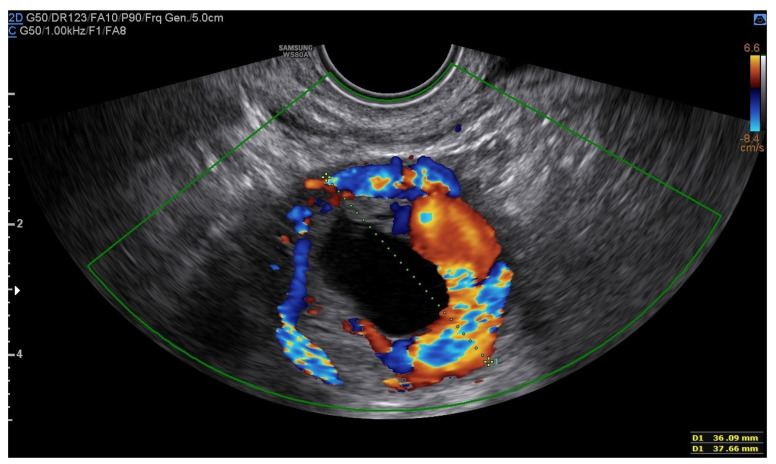
Arteriovenous malformation (AVM).

**Figure 4:**
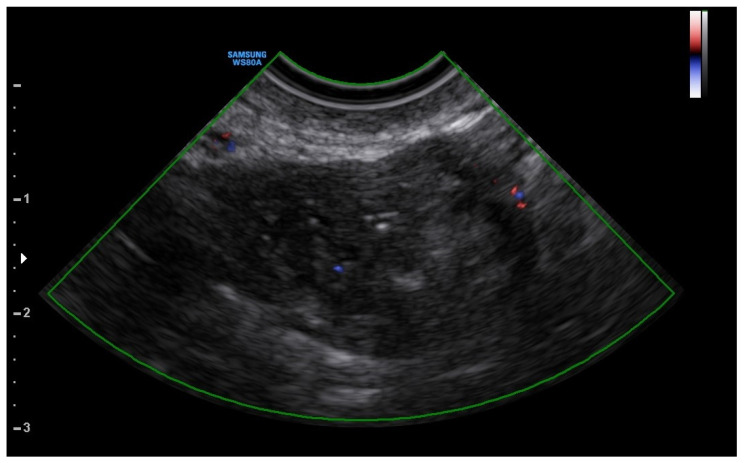
Resolution of the AVM after 3 months.

A 25-year-old woman, with a history of left tubal laparoscopic salpingectomy for an ectopic pregnancy, sought medical attention at the hospital’s emergency department after an episode of vaginal bleeding at 5 + 6 weeks of amenorrhea. At Day-1, her β-hCG was 2528 mUI/mL but a transvaginal ultrasound scan (TVUS) showed no clear evidence of pregnancy. Therefore, the patient was admitted with a diagnosis of pregnancy of unknown location (PUL). Upon admission, the patient was clinically and hemodynamically stable and did not report any pain or active bleeding. Serial measurement of her β-hCG in the following days was consistent with a suboptimal growth (3696 at Day-2, 5898 at Day-4, 10,984 at Day-7). Repeated TVUSs failed to show sure evidence of pregnancy until Day-7, when an intramural vascularized mass of 27 × 8 × 20 mm, with a gestational sac of 7 mm, a yolk sac and a 2 mm embryo with a heartbeat, was detected (Figure 1). Pelvic nuclear magnetic resonance (NMR) performed at Day-7 confirmed the presence of an intramyometrial T2-hypointense signal alteration of 20 mm in the right cornual area, surrounded by ectasic vessels, suggestive of interstitial pregnancy (Figure 2). After thorough counselling, a conservative management was proposed and accepted by the patient. The patient was administered a single oral dose of mifepristone 600 mg in combination with multidose systemic MTX 72 mg on days 0, 2, 4 and 6 from diagnosis. Follow-up by TVUS and β-hCG measurement was carried out. By Day-15, β-hCG reached a plateau and started reducing. A TVUS confirmed the termination of the pregnancy by involution of the gestational sac and the presence of the embryo with the disappearance of the fetal heartbeat. After 22 days from treatment, while the pregnancy was disappearing, in the same site, TVUS showed the appearance of a myometrial tubular hypoechoic region of 37 × 38 mm with an intensely vascular and multidirectional flow. Spectral Doppler ultrasound showed a low-resistance, high-velocity (peak systolic velocity of 128 cm/s) flow pattern, suggestive of uterine arteriovenous malformation (AVM) in the location of the previous interstitial pregnancy (Figure 3). The patient remained asymptomatic. To avoid menstrual bleeding and minimize the risk of metrorrhagia, a depot 3.75 mg shot of a GnRH analogue was administered every 28 days for 4 months. Considering the desire to preserve the fertility of the patient, the absence of guidelines for the treatment of AVM and the good results presented in the literature on uterine artery embolization as a conservative treatment, we performed a right uterine artery embolization, with sonographic resolution of the AVM within 3 months (Figure 4). We accessed the right common femoral artery and a 5-Fr introducing an angiographic sheath was placed. A 5-Fr Cobra catheter (Cook, Bloomington, IN, USA) was used to perform nonselective angiograms of the internal iliac arteries in order to achieve a general understanding of the vascular anatomy; the right internal iliac artery was selected after creating a Waltman loop with the Cobra catheter. The right uterine artery was selected using a microcatheter ranging from 2.0 to 2.4 Fr. As embolic materials, polyvinyl alcohol particles were used (Contour; Boston Scientific, Cork, Ireland). The rescue treatment had no complications. We present an unpublished case of interstitial pregnancy in a hemodynamically stable woman at an early gestational age successfully treated with medical therapy using MTX and mifepristone, who developed a peculiar complication.

## 3. Materials and Methods

A literature search was carried out in March 2023 using different combinations of the keywords “interstitial pregnancy”, “medical treatment”, “methotrexate” and “mifepristone”. Articles that were published in English from January 1991 until December 2022 were obtained from Scopus, PubMed and Google Scholar. The patient signed an informed consent form and gave permission for the publication of data in accordance with the 1964 Declaration of Helsinki and its later amendments. The Institutional Review Board (RC 08/2022) approved this descriptive study in April 2022.

Only articles in English were included in the search. The research strategy adopted included different combinations of the following terms: “interstitial pregnancy”, “medical treatment”, “methotrexate” and “mifepristone”.

**Figure 5:**
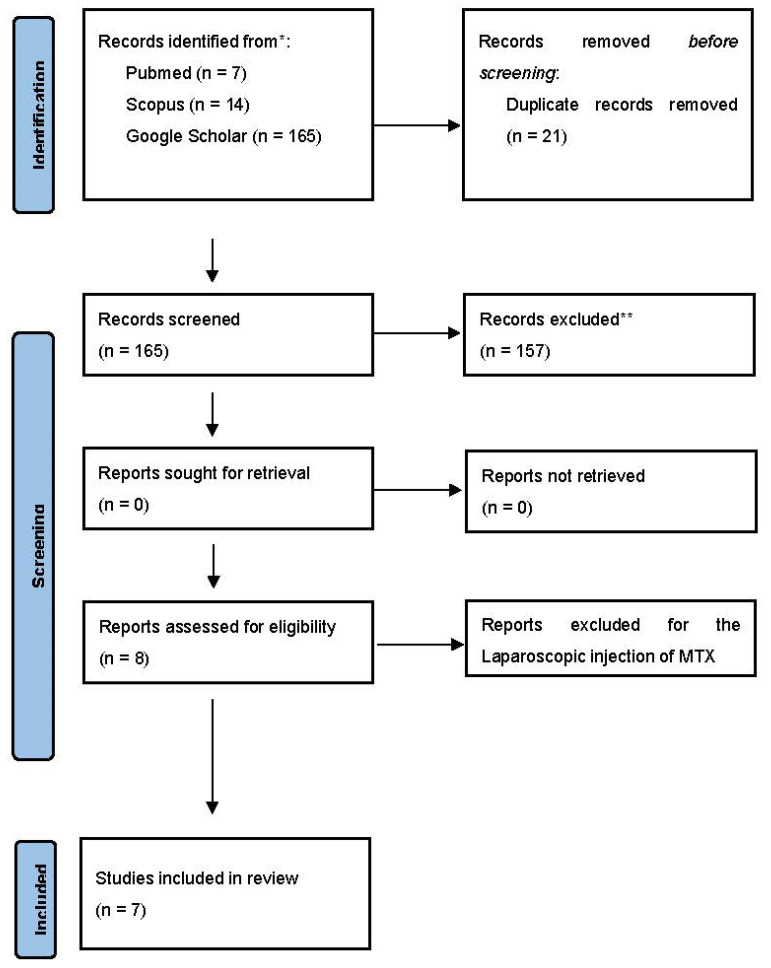
PRISMA flow diagram.

All studies identified were examined for their year, citation, title, authors, abstract and full texts. Duplicates were identified through manual screening performed by two researchers (G.S. and S.F.) and then removed. PRISMA guidelines were followed [15]. The PRISMA flow diagram of the selection process is provided in Figure 5. For the eligibility process, two authors independently screened the title and abstracts of all non-duplicated papers and excluded those not pertinent to the topic. The same two authors independently reviewed the full text of papers that passed the first screening and identified those to be included in the review. Discrepancies were resolved by consensus among the authors.

## 4. Results

We searched the literature with the aim of reviewing the effects associated with the reported cases of interstitial pregnancies treated using the combination of MTX with oral mifepristone in single or multiple systemic doses [4,11,12]. A total of 186 papers were found using the aforementioned keywords. After the exclusion of duplicates and studies that did not meet the inclusion criteria, eight papers were assessed for eligibility. One study was excluded because MTX was administered laparoscopically and not systemically [16]. Seven studies, which included 10 cases of interstitial pregnancies treated with systemic MTX + mifepristone, were considered eligible and included in the systematic review (Figure 5). To this analysis, we added our case reported in this paper. The total eleven patients’ clinical characteristics, ultrasound and laboratory findings, and type of treatment adopted are presented in Table 1.

In the sample, which included the present case, interstitial pregnancy was diagnosed at a mean gestational age of 6 weeks, with β-hCG levels ranging from 594 to 31.298 mIU/mL. The medium β-hCG level was 9933 mIU/mL. In 54.5% of cases (n = 6), an embryo was present inside the gestational sac. An embryonic heartbeat was detected in four of the six cases (66.7%). Six patients showed risk factors for ectopic pregnancy (54.5%): four patients had a previous salpingectomy for ectopic pregnancy in their medical history, while two had at least one previous dilatation and curettage (D&C) for voluntary interruption of pregnancy or spontaneous miscarriage. All patients were treated with a single dose of mifepristone, in combination with single or multiple doses of MTX i.m. In 7 cases out of 11 (63.6%), a single dose regimen was chosen, while in the remaining cases, a multidose strategy was used.

In 7 of the 11 cases reported (63.6%), the treatment with the combination of MTX and mifepristone led to the resolution of pregnancy with no side effects. One of the patients needed a second administration of the treatment as a second-line, with complete resolution. One was diagnosed with a hemoperitoneum and required laparoscopic salpingectomy with removal of the interstitial pregnancy. Another one had a second dose of MTX for incomplete resolution and then developed a hemoperitoneum, requiring surgical management by laparotomic cornuectomy with removal of the interstitial pregnancy. Lastly, our patient was diagnosed with a uterine arteriovenous malformation (AVM) after first-line treatment, which brought a significant reduction in β-hCG and pregnancy dissolution. We managed the complication conservatively through uterine artery embolization, accompanied by depot GnRH analogue administration to avoid excessive bleeding.

## 5. Discussion

Interstitial pregnancy is an uncommon complication of early pregnancy. It is associated with an increased risk of severe hemorrhage and maternal morbidity. Management of interstitial pregnancies remains a debated topic, with no clear guidelines on the best approach [20]. As interstitial pregnancy can occur in young women wishing to conceive again, conservative treatment has been proposed and has been demonstrated to be an effective option in select cases [12]. Expectant management could be considered after counseling with the patient, in cases where a reduction in β-hCG is detected, also considering that tubal ectopic pregnancies have resulted in significantly higher prospective spontaneous pregnancy rates when compared to salpingectomies [21].

Medical treatment for interstitial pregnancy includes the use of MTX, alone or in association with mifepristone.

MTX is a folic acid antimetabolite that has a high affinity for dihydrofolate reductase and binds to it in a competitive manner. It is currently recognized as the first-choice monotherapy for the conservative treatment of ectopic tubal pregnancy. MTX administration can be systemic (intramuscular or intravenous) or local (injection close or into the gestational sac, by laparoscopy, ultrasound or hysteroscopy guide). Different protocols have been used for MTX, the most common being single-dose, double-dose and multidose protocols [22].

The different protocols of MTX administration in ectopic pregnancy were compared by a systematic review and meta-analysis [23]. It was concluded that the overall success rate of the multidose protocol was similar to the single-dose protocol. These findings differ from those of a previous metanalysis, which favored the multidose regimen over the single-dose regimen, even if there were more reported side effects [24]. In the present case, due to the high level of β-hCG, the visualization of an embryo with a heartbeat and the presence of a rich vascularization of the lesion, we opted for a multidose regimen.

Mifepristone is a steroidal progesterone antagonist. Its ability to competitively combine with progesterone receptors and glucocorticoid receptors permits it to reduce the activity of progesterone, leading to cell degeneration and the demise of the decidua and chorion. It is routinely used to treat intrauterine pregnancy for interruption of pregnancy, and it has been proposed for the combined treatment of interstitial pregnancy [13].

Since mifepristone and MTX work differently in ectopic pregnancy, the combination of the two in the treatment of interstitial pregnancy can improve the cure rate more effectively, more significantly reduce β-hCG levels, improve symptoms such as vaginal bleeding and abdominal pain, and promote the absorption of the mass. Thus, combination therapy is deemed to increase clinical efficacy.

However, complications related to the use of the medical management of interstitial pregnancy were not examined in the literature.

We reviewed 11 cases of interstitial pregnancies treated with a combination of mifepristone and MTX in a single- or multidose regimen.

The systemic medical treatment was effective in 7 of the 11 cases.

In one case, β-hCG had an unexpected increase at 21 days from treatment after an initial drop, so the clinicians decided to administer a second injection of MTX. The complete negativization of β-hCG was achieved in 47 days [12].

Two cases of a hemoperitoneum following combined MTX and mifepristone treatment were reported. The first happened 10 days after administration, despite the reduction in size of the gestational sac at ultrasound and the decrease in β-hCG levels, suggesting a resolution of the pregnancy. It was managed laparoscopically through a right salpingectomy with removal of the interstitial pregnancy [19]. The second one was described in an interstitial pregnancy with high basal β-hCG (31.298 mUI/mL) with a 10 mm embryo with heartbeat. A second dose of MTX was needed because of a suboptimal decrease in β-hCG levels. After 20 days, hemoperitoneum occurred and was managed through a cornuectomy with removal of the interstitial pregnancy [10]. It is important to underline that the latter case was the one with the highest β-hCG among the cases presented and this could represent a reason for the failure of the treatment.

In our case, a multidose protocol of MTX combined with mifepristone was applied, with the apparent resolution of the interstitial pregnancy at ultrasound examination during follow-up. Nevertheless, during TVUS follow-up, we detected the appearance of an AVM of significant dimensions. To avoid excessive bleeding, we decided to administer a depot GnRH analogue until the complete resolution of the AVM, and we performed a uterine artery embolization as a conservative treatment for this complication. The use of uterine artery embolization under fluoroscopic and 3D ultrasound guidance could represent another strategy of treatment for interstitial pregnancies. However, the use of this treatment for this type of pathology has led to some cases of endometrial atrophy and adverse effects on the fertility of patients [25]. The impact on future fertility and pregnancy outcomes after uterine artery embolization was studied. It includes uterine blood flow impairment, which may compromise placental blood supply, and nontargeted embolization of the ovarian arteries, which can cause ovarian failure in perimenopausal women. However, a recent metanalysis reviewed the incidence and outcomes of pregnancies in patients treated with uterine artery embolization following AVM. It was concluded that uterine artery embolization is a viable option for women with AVM willing to conceive, as its impact on fertility does not appear to be substantial, and the outcome of pregnancy seems to be similar to that of the general population [26].

In our case, this technique was used to treat a complication derived from medical therapy and to avoid a more invasive surgery, with the execution of a cornuotomy. Our patient did not show signs of endometrial atrophy, reporting no symptoms and regular menstrual flows.

The strength of our study is the long period of time overviewed in the literature. We analyzed the cases of interstitial pregnancy treated with MTX and mifepristone from 1991. All the studies selected during the eligibility phase were further evaluated by manual comparison of populations, study settings and authors to avoid overlapping cases. The main limitation of this review is that only case reports were included among the papers selected, with this being due to the rarity of this complication.

## 6. Conclusions

The most appropriate management of interstitial pregnancy remains a debated topic, and guidelines about the best treatment to choose are lacking. The applicability of medical conservative treatment should be tailored to the patient, taking into account their obstetric history, gestational age at diagnosis, β-hCG levels and desire for future pregnancies. The use of a combination of systemic MTX in a single- or multidose regimen with mifepristone was found to be an effective option in 63.6% of cases. When this therapy achieved the complete resolution of the pregnancy, no major complications where described. The occurrence of pregnancy rupture with a hemoperitoneum should be considered and promptly treated surgically. The appearance of AVM can be managed conservatively, and we propose uterine artery embolization as an effective treatment of this rare complication.

## Figures and Tables

**Table 1:** Sample characteristics.

Case Reference	Pregnancy History	Gestational Age	CRL (mm)	FHB	β-hCG at Diagnosis	Management of IP	Complication	Management of Complication
Present case	G2P0 (1 previous salpingectomy for EP)	5 + 6	2	–	10,984	Mifepristone 600 mg + MTX 1 mg/kg on days 0, 2, 4 and 6 (multidose)	Uterine arteriovenous malformation (AVM)	Right uterine artery embolization and depot 3.75 mg shot of GnRH analogue every 28 days for 4 months
Stabile et al. 2021 [13]	G2P0 (1 previous salpingectomy for EP)	7	9	+	22,272	Mifepristone 600 mg + MTX 1 mg/kg on days 0, 2, 4 and 6 + 0.1 mg folinic acid (multidose)	None	
Stabile G. et al. 2020 [12]	G3P0 (2 previous D&Cs)	7	3.6	+	19,397	Mifepristone 600 mg + MTX 1 mg/kg on days 0 and 2 + 0.1 mg folinic acid (multidose)	None	
Stabile G. et al. 2020 [12]	G2P1	6 + 6	No embryo	–	2664	Mifepristone 600 mg + MTX 50 mg/m^2^ of body surface (single dose)	Need for repetition of dose	Second dose
Gomez Garcia et al. 2012 [9]	G2P1	8 + 3	No embryo	–	3724	Mifepristone 600 mg + IM MTX 50 mg/m^2^ of body surface (single dose)	None	
Gomez Garcia et al. 2012 [9]	G4P0 (3 previous voluntary interruptions of pregnancy)	6 + 3	6	–	4116	Mifepristone 600 mg + IM MTX 50 mg/m^2^ of body surface (single dose)	None	
Stabile G. et al. 2020 [17]	G3P1 (1 previous salpingectomy for EP)	6	No embryo	–	6579	Mifepristone 600 mg + IM MTX 50 mg/m^2^ of body surface (single dose)	None	
Stabile G. et al. 2020 [17]	G3P1	5 + 3	No embryo	–	2124	Mifepristone 600 mg + IM MTX 50 mg/m^2^ of body surface (single dose)	None	
Restaino et al. 2022 [18]	G3P1 (1 previous salpingectomy for EP)	6	5.7	+	5520	Mifepristone 600 mg + IM MTX 50 mg/m^2^ of body surface (double dose)	Hemoperitoneum	Laparoscopy: right salpingectomy with removal of the interstitial pregnancy
Sorrentino et al. 2022 [10]	G1P0	6 + 5	5.5	+	31,298	Mifepristone 600 mg + IM MTX 50 mg/m^2^ of body surface (single dose)	Need for repetition of dose, hemoperitoneum	Second dose, laparotomy: cornuectomy with removal of the interstitial pregnancy
Karki et al. 2015 [19]	G1P0	5 + 1	No embryo	–	594	Oral mifepristone 200 mg + IM MTX 50 mg/m^2^ of body surface (single dose)	None	

## Data Availability

The authors confirm that the data supporting the findings of this study are available within the article.
